# The Role of Hepatocellular Carcinoma Surveillance in Autoimmune Hepatitis

**DOI:** 10.7759/cureus.11269

**Published:** 2020-10-30

**Authors:** Ahila Manivannan, Samia Mazumder, Nabil Al-Kourainy

**Affiliations:** 1 Internal Medicine, Wayne State University School of Medicine, Detroit, USA

**Keywords:** hepatocellular carcinoma (hcc), autoimmune hepatitis, screening guidelines

## Abstract

Type 1 autoimmune hepatitis (AIH) is a rare inflammatory disorder of the liver that may arise at any age, from infancy to adulthood. Long-standing autoimmune hepatitis may progress to cirrhosis and subsequent hepatocellular carcinoma (HCC). However, the true incidence of HCC in AIH patients is unknown as there is a paucity of published data. Currently, there are no established guidelines on screening patients with AIH for HCC. Without screening protocols, patients with AIH may present with late-stage HCC that may have been detected and treated earlier in the disease course. We describe a case of a patient with type 1 AIH who developed stage IIIB HCC in the absence of appropriate screening protocols with complex social determinants leading to barriers to access regular follow-up care.

## Introduction

Autoimmune hepatitis (AIH) is a disorder that is primarily characterized as chronic inflammation of the liver. Although the etiology of AIH is unknown and variable, several studies suggest that it may be environmentally triggered. This process results in the development and subsequent loss of tolerance to neoantigens that incite an inflammatory response throughout the hepatic system [1]. The worldwide prevalence of AIH is estimated to range between four and 25 per 100,000 individuals and is four times as likely to occur in females [2]. 

AIH can be further classified into two subcategories, type 1 and type 2, determined by the type of circulating autoantibodies. Antinuclear antibody, anti-smooth muscle antibody, and anti-liver-kidney microsomal-1 antibodies are characteristic of type 1 AIH. Type 2 AIH is characterized by anti-liver cytosol antibody-1 or anti-liver-kidney-microsomal-1 antibody alone [3]. Although antibody profiles may be used to categorize patients with AIH, some patients may not have circulating autoantibodies at all. In these cases, AIH may be diagnosed based on clinical presentation, liver histology, and the presence of IgG greater than the upper limit of normal [4,5].

Patients with AIH may present with a variety of clinical symptoms, ranging from an asymptomatic elevation in liver enzymes to acute liver failure. Clinical features of AIH include elevated liver enzymes (aspartate aminotransferase or alanine aminotransferase), increased IgG or gamma-globulin levels or the presence of autoantibodies [6]. AIH is often a diagnosis of exclusion. Therefore, it is important to exclude other diseases that may present with a similar clinical picture, such as primary biliary cholangitis, viral hepatitis, drug-induced liver injury, or other autoimmune diseases, such as lupus-associated liver disease. 

One of the most significant complications of AIH is cirrhosis and subsequent hepatocellular carcinoma (HCC). The risk of developing HCC in patients with AIH has historically been assumed to be low. A systematic review by Tansel et al. demonstrated that while the risk of HCC in AIH patients appears lower than that of hepatitis B, C, or primary biliary cholangitis patients, 92 out of the 93 AIH patients had cirrhosis by the time of the HCC diagnosis [7]. Therefore, it is important to understand the association between AIH and cirrhosis when treating patients with AIH. 

Hepatocellular carcinoma is one of the top causes of cancer-related death in the United States. The incidence of HCC has tripled since the 1980s and the five-year survival rate is 18%. If HCC is not detected in a timely manner before metastasis, the five-year survival rate drops to 2% [8,9]. The current screening guidelines for HCC recommend an alpha-fetoprotein (AFP) level and abdominal ultrasound every six months. These recommendations are primarily centered around patients that are classified as “high-risk”, such as those with active hepatitis, a family history of HCC, and primary liver cirrhosis. However, there are no established guidelines for screening patients with AIH prior to the development of cirrhosis and subsequent HCC. 

We present a case of a patient with type 1 AIH who developed HCC 15 years after the initial diagnosis. This case highlights an important association between type 1 AIH and HCC. In addition, this case suggests a role for early screening in patients with AIH in order to prevent the progression of cirrhosis to HCC. 

## Case presentation

A 35-year-old African American male with a 20-year history of type 1 AIH, liver cirrhosis, primary sclerosing cholangitis, and esophageal varices presented with a two-day history of confusion and bizarre behavior. He had been recently diagnosed with HCC and was taking lactulose and lenvatinib 12 mg daily two weeks prior to presentation. He was not able to titrate his bowel movements with lactulose at home due to chemotherapy-induced diarrhea, which resulted in missing two doses of lactulose. The patient was subsequently diagnosed with hepatic encephalopathy secondary to lactulose non-adherence in the setting of chemotherapy-induced diarrhea. During his hospital course, the patient was treated with lactulose and rifaximin, which resolved his encephalopathic symptoms. He is currently being evaluated for a liver transplant and is being monitored closely by his oncology team at a tertiary care center. 

Case background

The patient was diagnosed with type 1 AIH at the age of 15 following a liver biopsy, which showed cirrhosis and inflammatory activity consistent with AIH. To manage recurrent AIH episodes, he was prescribed azathioprine, prednisone, and propranolol by a gastroenterologist at a tertiary care hospital. Unfortunately, due to complex social determinants of health including insurance coverage issues and unwanted medication side effects, he was mostly non-adherent to the management plan. Due to the patient’s multiple gastrointestinal comorbidities, a series of surveillance tests were prescribed in 2013, including an outpatient MRI, AFP monitoring, and surveillance abdominal ultrasounds every six months to screen for cholangiocarcinoma and primary liver cancer. However, insurance coverage challenges prevented the patient from maintaining consistent follow-up.

**Figure 1:**
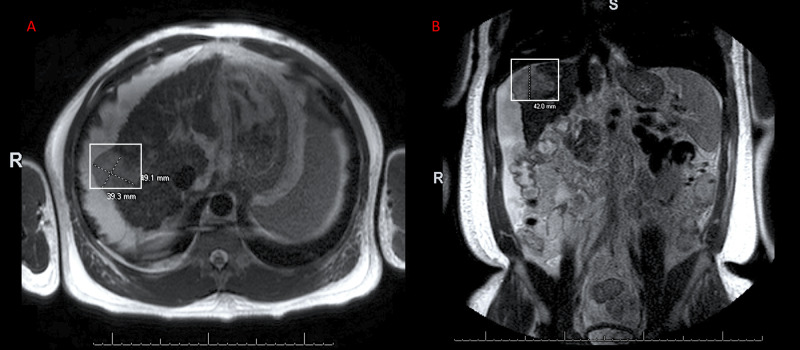
Exophytic Hepatic Mass Suggesting Hepatocellular Carcinoma Seen in MRI of the Abdomen with Contrast MRI (magnetic resonance imaging) of the abdomen with contrast demonstrates an exophytic mass, which measured 3.93 by 4.91 mm in the transverse view (Panel A) and 4.20 mm in the coronal view (Panel B).

Approximately one month prior to his hospital presentation with hepatic encephalopathy, he was diagnosed with HCC. This was determined after a workup for presentation with bilateral lower extremity and testicular swelling yielded the presence of a hepatic mass (4.91 by 3.93 mm) seen on MRI with a right portal vein thrombus (Figure 1).

**Figure 2:**
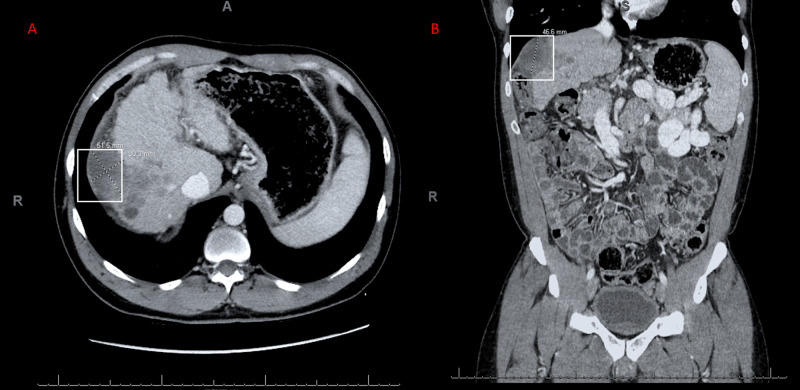
Growth of Exophytic Hepatic Mass Seen In CT of the Abdomen with Contrast One month after the initial diagnosis for HCC (hepatocellular carcinoma), CT (computerized tomography) of the abdomen with contrast demonstrated a growth in the exophytic mass. The mass measured 5.15 by 3.83 mm in the transverse view (Panel A) and 46.6 mm in the coronal view (Panel B).

Laboratory studies were notable for elevations in AFP (8418.2 ng/mL) and bilirubin (6.7 mg/dL). Subsequent percutaneous needle biopsy of the liver confirmed the diagnosis of well-differentiated HCC, with biopsy positive for arginase-1 and negative for cytokeratin-7. Subsequent CT of the chest, abdomen, and pelvis with contrast for staging was performed one month after the initial diagnosis. While it demonstrated no evidence of metastatic disease, it did reveal progression of the hepatic mass to 5.15 by 3.83 mm (previously 4.91 by 3.93 mm) along with central invasion of the tumor (Figure 2).

With these findings, our patient was diagnosed with stage IIIB HCC.

## Discussion

Our case highlights the importance of establishing standardized HCC screening guidelines for patients with AIH prior to the development of cirrhosis. Screening protocols for HCC in patients with AIH should utilize low cost, non-invasive diagnostic strategies with high specificity, such as abdominal ultrasound and serum biomarkers. Despite the known benefits, these screening modalities are currently underutilized [10]. While AFP monitoring improves the sensitivity of HCC detection, more reliable serum biomarkers should be investigated [10]. The optimal AFP threshold remains a topic of debate and our case reflects the current threshold for clinicians to consider is between 200 to 400 ng/ml [11].

In the case of our patient, a streamlined screening protocol may have been critical to ensure consistent follow-up and adequate insurance coverage. Low adherence to existing HCC surveillance protocols may disproportionately affect minorities and patients of low socioeconomic status, similar to our patient [10]. As illustrated in the case above, pediatric African American patients diagnosed with AIH are more likely to present with end-stage liver disease, undergo a liver transplant, and experience disease recurrence after transplant compared to non-African American pediatric patients [12]. Further studies are warranted to delve deeper into the particular biological and socioeconomic factors that may lead to worsened outcomes in African American patients in particular. It is imperative to target patients with poor prognostic factors in order to treat them with tailored immunosuppressive therapy, which our patient did not receive. Additionally, these patients should be referred to a transplant center early in their disease course in order to prevent progression to late-stage HCC. 

It is also important to note that our patient’s tumor increased in size within one month while being on lenvatinib therapy. Lenvatinib is a tyrosine kinase inhibitor that is a first-line option in adult patients who have advanced or unresectable HCC without prior systemic treatment. Lenvatinib has demonstrated a higher response rate and longer progression-free survival than an older first line drug, sorafenib [13]. This suggests that HCC with underlying AIH may require more aggressive treatment early on.

Our case suggests that established protocols for HCC and cirrhosis surveillance should be implemented early in the disease process of AIH. Patients should be stratified by risk factors such as early onset of AIH, number of relapses, development of cirrhosis or primary biliary cholangitis, and barriers to healthcare access. The efficacy of surveillance for HCC in patients with AIH should also be more thoroughly studied. Yeoman et al. found that the median survival in patients whose HCC was diagnosed on surveillance was significantly higher compared with patients presenting symptomatically (19 months versus two months) [14]. This further emphasizes the need for standardized, accessible surveillance for HCC in AIH patients.

## Conclusions

We presented a case of HCC in the setting of a 20-year history of AIH with multiple complications. The patient failed to receive an individualized treatment regimen despite establishing care with a tertiary care hospital with a transplant center prior to his HCC diagnosis. Our patient’s barriers to healthcare access also contributed to the challenges he experienced while attempting to adhere to the extensive screening regiment prescribed to him. This case highlights the need for standardized screening guidelines for patients with AIH stratified by risk factors specific to the disease process. Additionally, clinicians should be aware of the complicated progression of this disease and mindful of the challenges patients may face in adhering to necessary surveillance screenings for HCC, due to complex social determinants of health. 
